# Supplementary material for: Dietary Supplementation of a New Probiotic Compound Improves the Growth Performance and Health of Broilers by Altering the Composition of Cecal Microflora
Source: Biology (Basel). 2022 Apr 21;11(5):633. doi: 10.3390/biology11050633 (PMC9138300; doi:10.3390/biology11050633)
Supplement: Supplementary file 1 [file biology-11-00633-s001.zip › biology-1643448-supplementary.pdf]

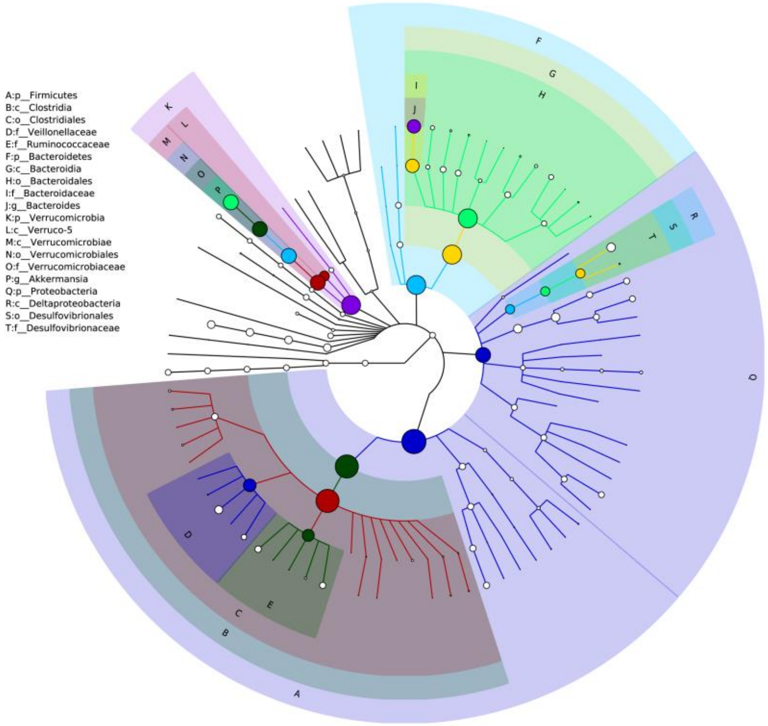

Figure S1. Linear discriminant analysis effect size (LEfSE) identified the most differentially abundant taxa enriched in cecal microbiota of broilers

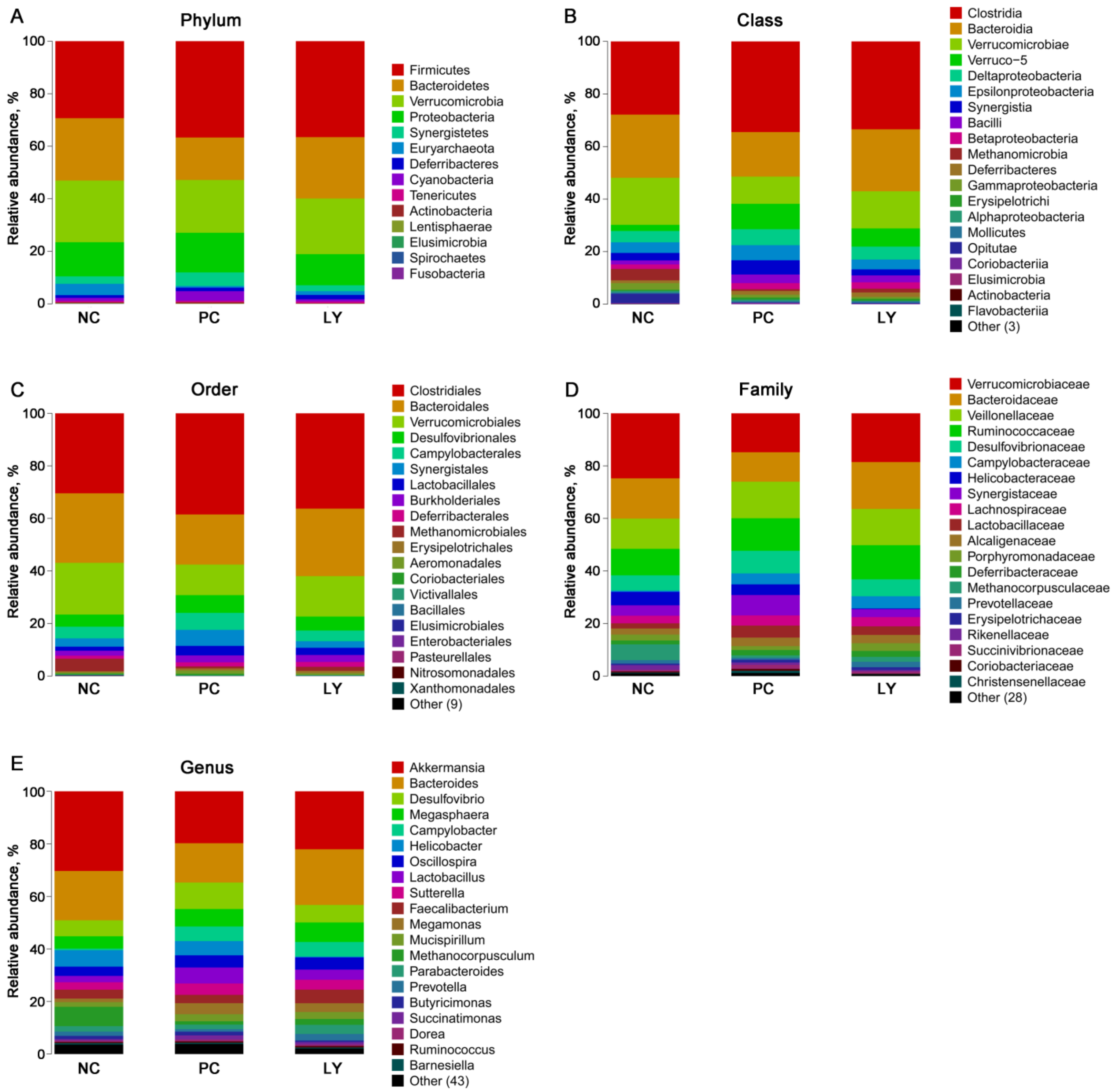

Figure S2. Bacterial community compositions of cecal microbiota of broilers at phylum (A), class (B), order (C), family (D) and genus (E) level.
